# A cross-sectional study of the prevalence of lameness and digital dermatitis in dairy cattle herds in Egypt

**DOI:** 10.1186/s12917-023-03620-5

**Published:** 2023-05-05

**Authors:** Shebl E. Salem, Ayman Mesalam, Ahmed Monir

**Affiliations:** 1 Department of Surgery, Anaesthesiology, and Radiology, Faculty of Veterinary Medicine, Zagazig University, Zagazig, 44519 Egypt; 2 Department of Theriogenology, Faculty of Veterinary Medicine, Zagazig University, Zagazig, 44519 Egypt

**Keywords:** Dairy, Cattle, Egypt, Lameness, Locomotion score, Digital Dermatitis, Hock score, Cow hygiene

## Abstract

**Background:**

Lameness is a significant problem for the dairy industry worldwide. No previous studies have evaluated the prevalence of lameness or digital dermatitis (DD) in dairy cattle herds in Egypt. A total of 16,098 dairy cows from 55 dairy herds in 11 Egyptian governorates underwent visual locomotion scoring using a 4-point scoring system. Cows that had a lameness score ≥ 2 were considered clinically lame. Following manure removal with water and using a flashlight, the cows’ hind feet were examined in the milking parlour to identify DD lesions and classify with M-score. Furthermore, each cow was assigned a hock score (a 3-point scale) and a hygiene score (a 4-point scale). The cow-, within-and between-herd prevalence of lameness and DD and associated 95% confidence intervals (CI) were calculated. The prevalence of hock lesions and poor cow hygiene was also calculated.

**Results:**

Of the examined cows, 6,883 were found to be clinically lame (42.8%, 95% CI = 42.0–43.5%). The average within-herd prevalence of lameness was 43.1% (95% CI = 35.9–50.3%). None of the dairy herds recruited into the study were found to be free from clinical lameness. The average within-herd prevalence of DD was 6.4% (95% CI = 4.9–8.0%). The herd-level prevalence of DD was 92.7% (95% CI = 85.9–99.6%). Active DD lesions (M1, M2, M4.1) were identified in 464 cows (2.9%) while inactive lesions (M3, M4) were identified in 559 cows (3.5%). The within-herd prevalence of hock lesions (score 2 or 3) was 12.6% (95% CI = 4.03–21.1%) while a severe hock lesion had within-herd prevalence of 0.31% (95% CI = 0.12–0.51%). Cow-level prevalence of hock lesions was 6.2% (n = 847, 95% CI = 5.8–6.2%). The majority of examined cows had a hygiene score of 4 (n = 10,814, prevalence = 70.3%, 95% CI = 69.5–71%).

**Conclusions:**

The prevalence of lameness was higher than prevalence estimates reported for other countries which could be due to differing management and/or environmental factors. DD was identified at lower prevalence in most herds but with high herd-level prevalence. Poor cow hygiene was notable in most herds. Measures to reduce the prevalence of lameness and to improve cow hygiene in dairy cattle herds in Egypt are therefore needed.

## Background

Lameness is a major welfare and economic concern of the dairy industry worldwide [1]. Lameness is defined as clinical signs of impaired locomotion mostly due to lesions in the feet of the hind limbs [2]. Economic losses associated with lameness have been recently reviewed and classified as additional costs due to treatment and investment in prevention, losses due to reduced milk production, discarded milk due to treatment with antibiotics, reduced reproductive performance and increased culling rates and herd depreciation costs [3]. Lame cows have been reported to produce a lower cumulative milk yield compared with non-lame cows and are more likely to be culled from the herd [1, 4]. Furthermore, lameness diagnosed at drying-off has been found to be associated with transition period diseases such as hypocalcaemia, displaced abomasum, and metritis [5]. Lameness has also been associated with marked behaviour changes of cows, including feeding and laying behaviours [6–8].

Knowledge of the herd-level prevalence of lameness is key to estimate the impact of the disease on the industry and to evaluate the usefulness of strategies to reduce lameness [9]. Furthermore, prompt detection and treatment of lame cows can result in reduced duration and prevalence of lameness and improved production and welfare outcomes [10, 11]. A recent systematic review and meta-analysis from the UK reported a pooled lameness prevalence in British dairy cattle of 29.5% (95% CI 26.7–32.4%) and a pooled incidence rate of 30.9 cases of lameness per 100 cow-years (95% CI 24.5–37.9) [12]. An average lameness prevalence of 21–24.6% has been reported in North American dairy herds housed in free-stall barns [13–15]. In addition, many studies have reported on the prevalence of various foot lesions identified during routine hoof trimming [16–18]. To our knowledge, there are no previous studies that have investigated the prevalence of lameness in dairy cattle herds in Egypt. Such a study is important to quantify the impact of lameness on dairy herds in Egypt and to evaluate any future interventions to reduce lameness.

Digital dermatitis (DD) is an infectious skin disease of the foot that is characterised by painful ulcerative or hyperkeratotic lesions [19]. It was first identified in dairy herds in Italy in the 1970s and has become endemic in dairy herds worldwide since then, with variable prevalence being reported [20]. Although the presence of DD is not always associated with altered locomotion, studies have found that cattle identified with DD lesions were 8 and 10 times more likely to be diagnosed as either lame or moderately to severely lame compared with cattle without lesions, respectively [21]. DD was found to be the most treated foot lesion by hoof care professionals in the USA in 2017 [22]. The prevalence and impact of DD in Egyptian dairy herds are yet to be elucidated. To date, only one study has investigated the prevalence of DD in a single dairy herd in Egypt and found that DD had a 12-month cumulative incidence of 33% [23]. As the previous study was only conducted in a single dairy herd, the results could not be generalized to other dairy herds in Egypt.

The objectives of the current study were to determine baseline prevalence (cow-, within- and between-herd prevalence) of lameness using visual mobility scoring on a sample of dairy cattle herds in Egypt, and to determine the prevalence of DD lesions through examination of cows’ hind feet in the milking parlour. Additionally, the prevalence of hock lesions and the level of cow hygiene were evaluated.

## Results

**Fig. 1:**
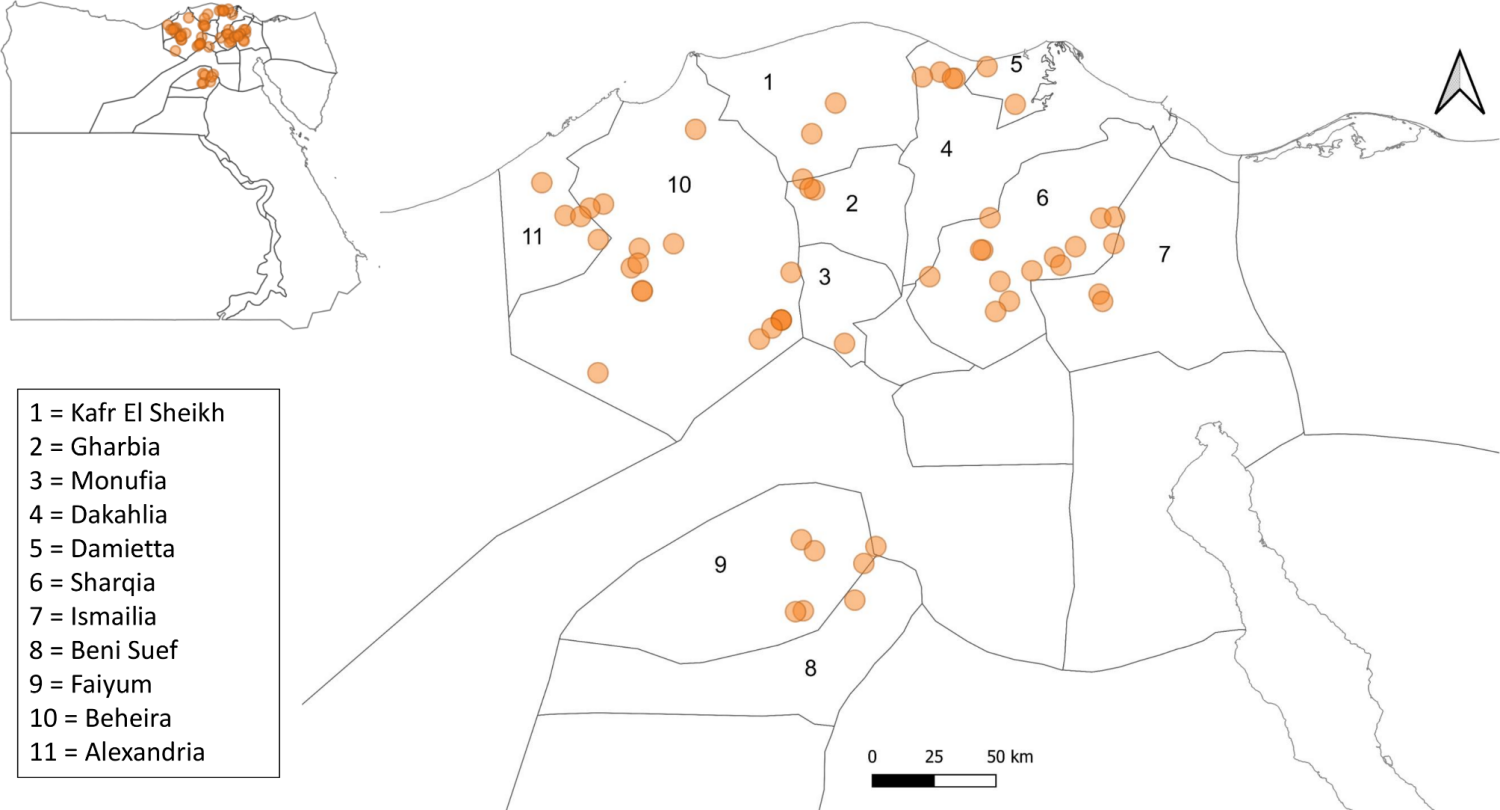
Approximate locations of 55 dairy farms included in the study. Each orange dot represents an approximate farm location

The owners/managers of 55 dairy farms consented to participate in the study. Farm visits were conducted between 9 April and 30 September 2022. Locations of the visited farms spanned 11 different Egyptian governorates (Kafr El Sheikh, Gharbia, Monufia, Dakahlia, Damietta, Sharqia, Ismailia, Beni Suef, Faiyum, Beheira, Alexandria). Figure 1 shows the approximate locations of visited farms. The median number of milking cows examined per farm was 191 cows (range 50–1,705 cows, interquartile range [IQR] 115, 322 cows). All visited farms kept cattle in open yards, most of which had sand bedding and shades that covered around 50% of the area of the yards. The yards were fitted with fans and water sprinklers on most farms. One or two milking parlours were installed on each farm according to the size of the herd. A single farm contained a closed free-stall barn that accommodated around 500 milking cows. Cows were fed a total mixed ration (TMR) that was formulated and distributed according to the stage and level of milk production. The TMR was composed mainly of corn silage, corn, soya bean and mineral and vitamin mixtures.

**Fig. 2:**
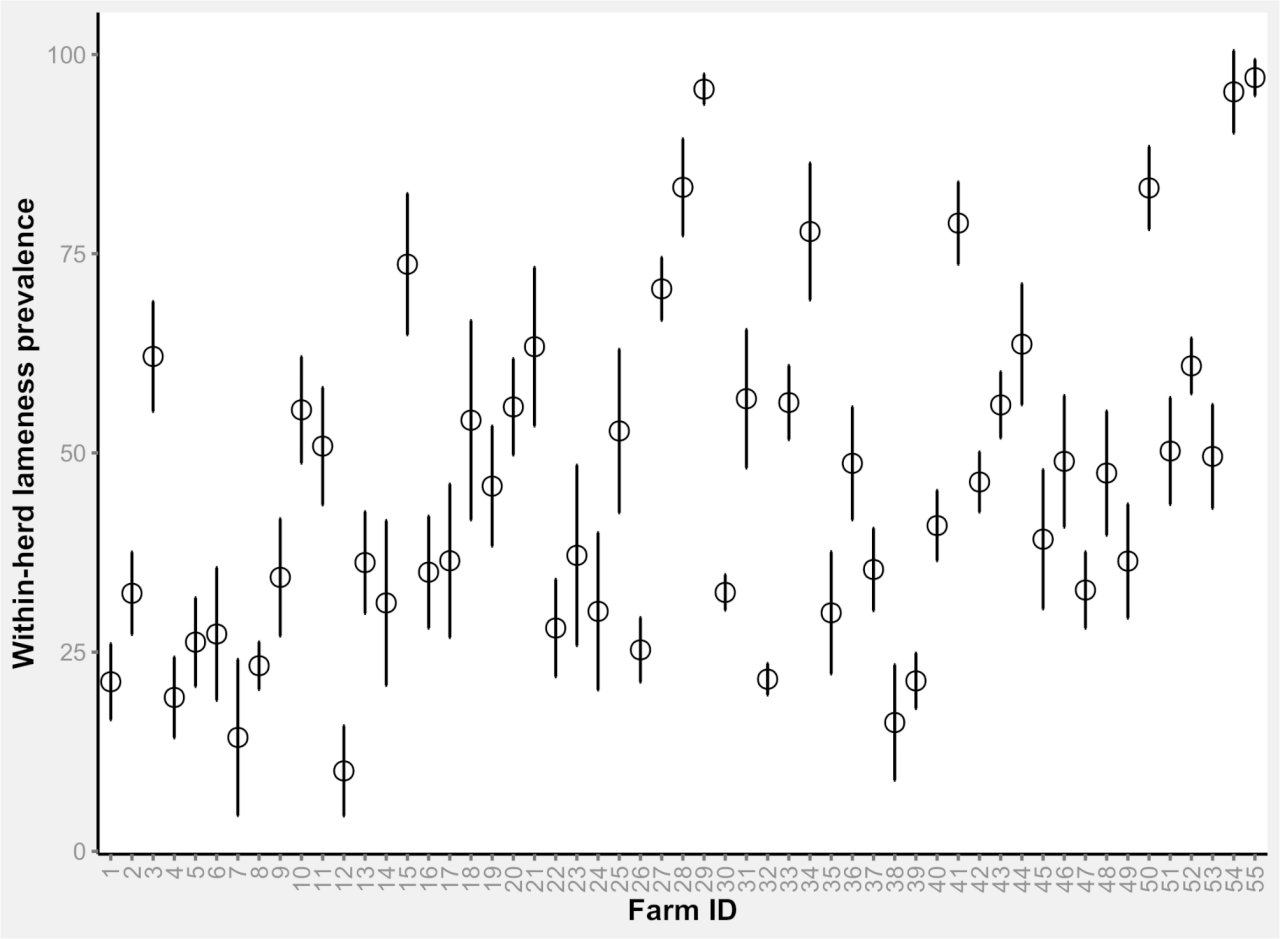
Prevalence and associated 95% Wald confidence intervals of clinical lameness (mobility score ≥ 2) in 55 dairy farms in Egypt. The circles represent prevalence and bars represent the lower and upper 95% Wald confidence intervals

The mean within-herd lameness prevalence (cows scored ≥ 2) adjusted for clustering within herds was 43.1% (range 10.1–97.1%, 95% confidence interval [CI] = 35.9–50.3%). Lameness prevalence and associated 95% Wald CI on each of the visited farms is provided in Fig. 2. The within-herd lameness prevalence was < 25% in 8 dairy herds, from 25% to < 50% in 25 dairy herds, and ≥ 50% in 22 dairy herds. A total of 16,098 cows underwent mobility scoring, of which 6,883 cows were found to be clinically lame (42.8%, 95% CI = 42–43.5%), 4,314 cows were scored 2 (26.8%, 95% = CI 26.1–27.5%) and 1,852 cows were scored 3 (11.5%, 95 CI = 11–12%). Distinction between a mobility score of 2 and 3 was not performed in 6 farms.

**Fig. 3:**
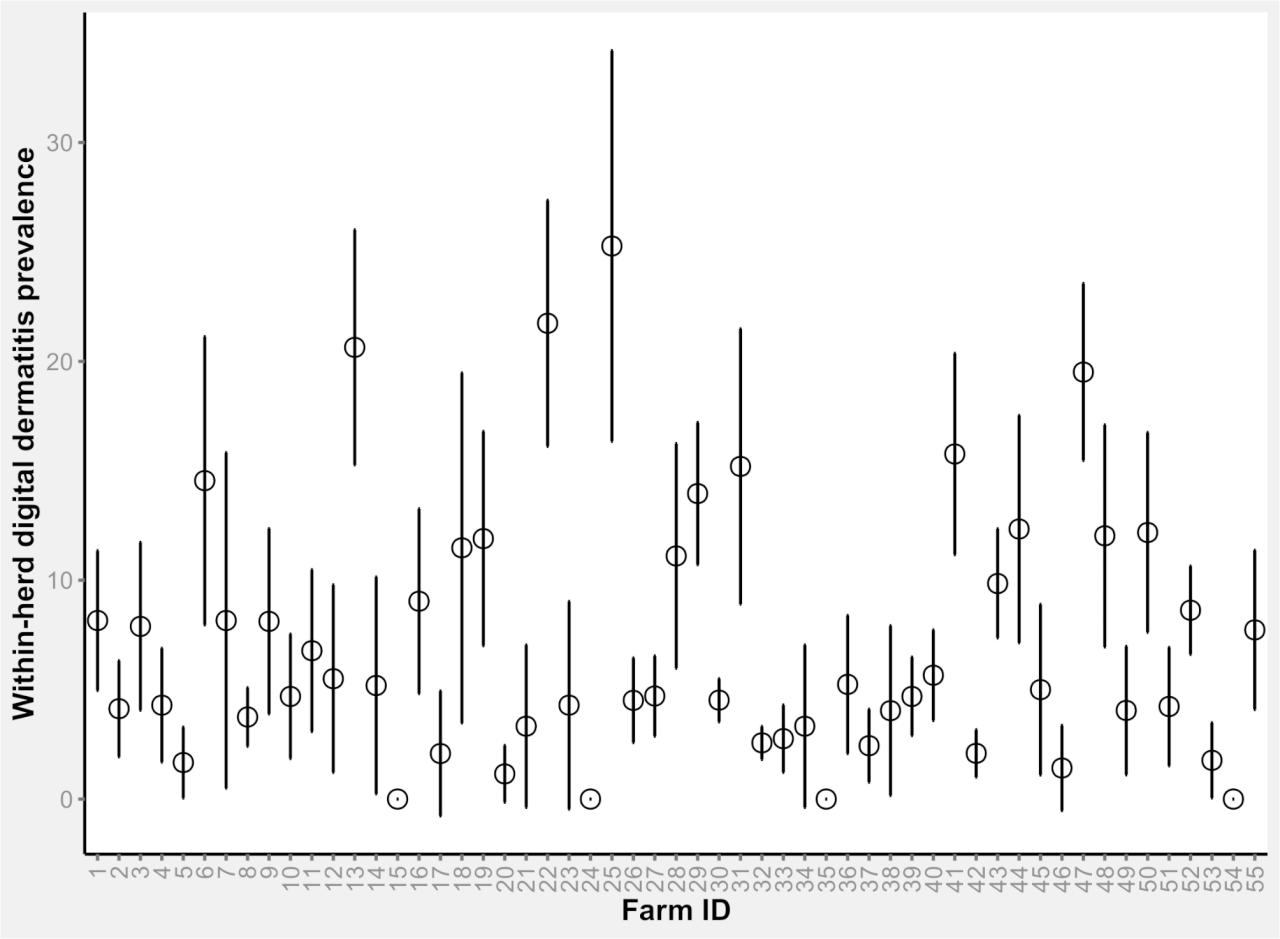
Within-herd prevalence of digital dermatitis in 55 dairy cattle herds in Egypt. The circles represent prevalence and bars represent the lower and upper 95% Wald confidence intervals

The area of the digital skin of the hind feet was examined with the help of a flashlight following manure removal with water to diagnose DD lesions. The mean within-herd prevalence of DD, adjusted for clustering within herds, was 6.4% (range 0–25.3%, 95% CI = 4.9–8.0%). Between-herd prevalence of DD was 92.7% (n = 51, 95% CI = 85.9–99.6%); four farms were DD-negative. In addition, active lesions (M1, M2, M4.1) were not identified in another 10 dairy herds. Figure 3 shows within-herd prevalence of DD and associated 95% Wald CIs on the visited farms. Of the examined cows, 1,023 were DD-positive (6.4%, 95% CI = 6.0–6.7%). Active DD lesions (M1, M2, M4.1) were diagnosed in 464 cows (2.9%, 95% CI = 2.6, 3.1%) while inactive/chronic lesions (M3, M4) were diagnosed in 559 cows (3.5%, 95% CI = 3.2–3.8). The majority of DD-positive farms (n = 37) had DD lesions in < 10% of examined cows, 11 farms had DD prevalence between 10% and < 20%, and only 3 farms had DD lesions in ≥ 20% of the examined cows.

**Fig. 4:**
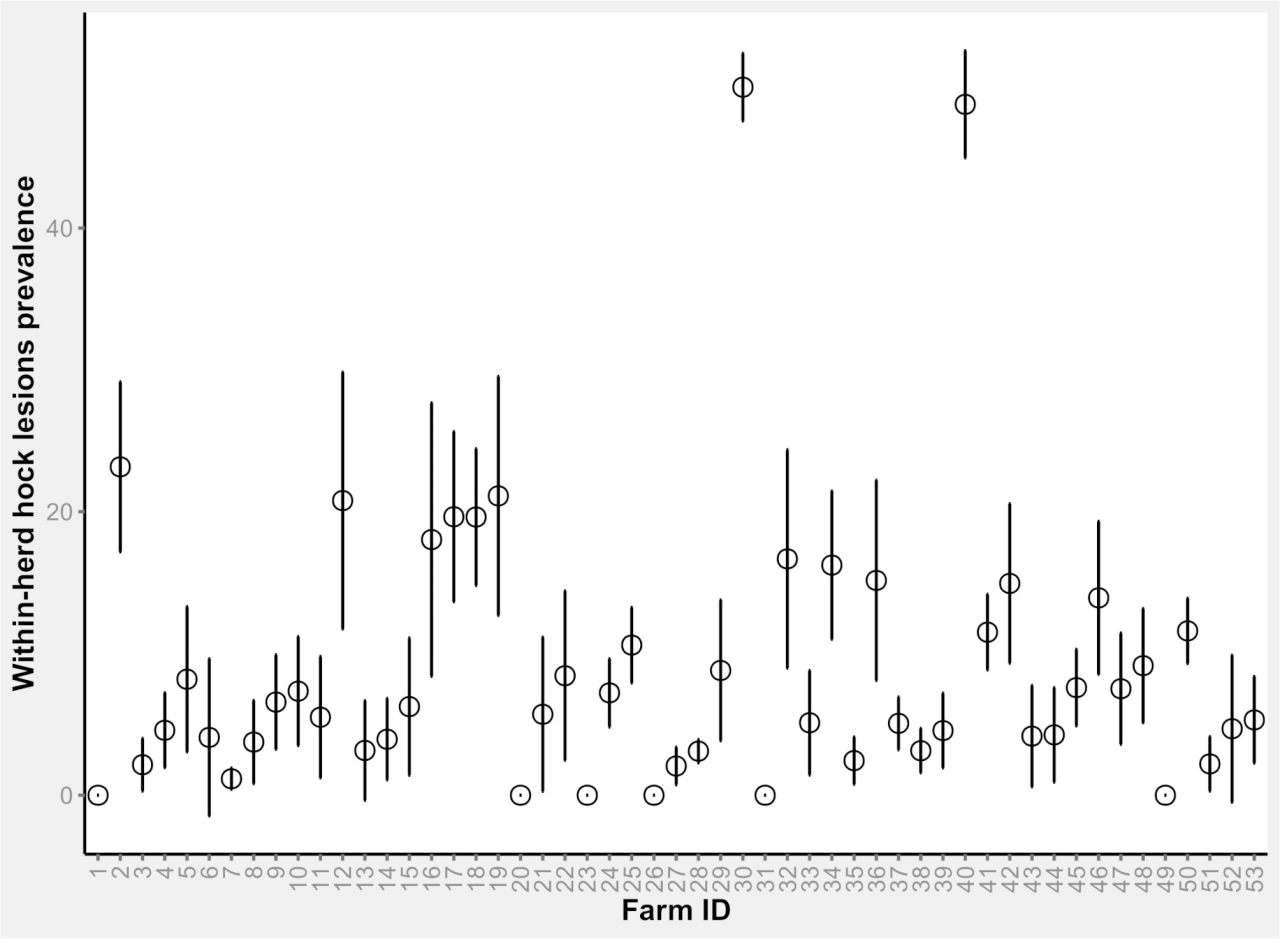
Within-herd prevalence of hock lesions on 53 dairy cattle herds in Egypt. The circles represent prevalence and bars represent the lower and upper 95% Wald confidence intervals

The average within-herd prevalence of hock lesions, adjusted for clustering within herds, was 12.6% (range 0, 49.9%, 95% CI = 4.03–21.1%). The prevalence of hock lesions was < 10% in 37 herds, between 10% and < 20% in 11 herds and ≥ 20% in 5 herds (Fig. 4). Severe hock lesions (hock score = 3) had a within-herd prevalence of 0.31% (95% bootstrap CI = 0.12–0.51). The cow-level prevalence of hock lesions was 12.9% (n = 2,011, 95% CI = 12.4–13.4%). Two large dairy herds had hock lesions in almost 50% of their lactating cows. One of these herds had a closed free-stall barn fitted with cubicles and rubber mattresses and the other farm had loose housing barns that had a clay soil with insufficient bedding. Removal of these two herds resulted in a reduction in within-herd prevalence to 6.4% (95% bootstrap CI = 4.7–8.2) and in cow-level prevalence to 6.2% (n = 847, 95% CI = 5.8–6.2%). The herd-level prevalence of hock lesions was 88.7% (n = 47, 95% CI = 80.2–97.2%). Hock lesions were not evaluated in two herds.

The cow-level prevalence of hygiene score > 2 was 91.3% (n = 14,056, 95% CI = 90.9–91.8%). A hygiene score of 4 was evident in most cows (n = 10,814, prevalence = 70.3%, 95% CI = 69.5–71%). Cow hygiene was not evaluated in 3 herds. The within-herd prevalence of a cow hygiene score of 3 or 4 was 91.2% (95% bootstrap CI = 86.1–96.3%).

## Discussion

Cattle and buffalo livestock production (dairy and meat) represents about 23% of total agricultural value in Egypt [24]. The bovine production system is highly heterogenous, consisting of large specialised production, small-scale farms, and household livestock production. The intensive bovine production system represents 7% of the total bovine population in Egypt [24] and these are mostly Holstein cattle originally imported from North America and Europe. Egypt produces around 3.5 million tons of raw milk from cattle, representing approximately 64% of total raw milk production [25]. Despite the importance of lameness as a welfare and economic problem in dairy cattle, no previous studies have evaluated the prevalence or the impact of lameness on the Egyptian dairy industry.

The average within-herd lameness prevalence reported in the present study (43.1%) was greater than the previously reported prevalence in other countries and regions such as the UK (29.5%) [12], North America (21–24%) [13–15], and Australia (18.9%) [26]. Differences in prevalence estimates could be due to variation in management practices; in our study population, all cattle were kept in loose-housing barns, compared with free-stall and tie-stall management systems practiced in the UK [27] and North America [13], and pasture-based housing in Australia [26]. A recent study reported that a farm profile characterised by exposure to high yearly temperature and humidity, with an open yard housing system and use of total mixed ration composed mainly of corn silage year around was associated with a higher disease risk for anoestrous, lameness, acute mastitis, and ovarian cysts compared with other farm profiles [28]. This farm profile closely resembles the management practices of dairy cattle herds in Egypt and could explain the reason for greater lameness prevalence reported in the present study.

High temperature humidity index (THI) was reported to be associated with reduced reproductive performance in multiple studies conducted in Egypt [29, 30]. Furthermore, heat stress has been associated with increased standing time, and decreased lying time and walking activity [31] which might increase the risk for lameness [32, 33]. The present study was conducted in the months of April to September, which coincides with the period of the greatest THI (≥ 75) in Egypt [34], and this might partially explain the greater lameness prevalence reported in the present study.

The greater lameness prevalence reported in the present study could also be due to lack of practising routine lameness preventive strategies on the visited farms such as routine hoof trimming, routine mobility scoring and foot-bathing which have been frequently reported to be associated with a reduction in the risk of lameness [10, 11, 35, 36]. Information about lameness preventive strategies has been collected from the participating farms and will be reported separately. The lameness prevalence reported in the present study also suggests that preventive strategies could have greater influence in reducing lameness risk in the studied population of dairy cattle.

Several studies from different countries have reported on the herd- and cow-level prevalence of DD. Yang et al. [37] surveyed 224 dairy herds in New Zealand through examination of cows’ hind feet in the milking parlour and reported a herd-level prevalence of 63.8%, and a mean within-farm prevalence of < 3% on around half of the farms. The maximum within-farm prevalence was 12.7% and the overall cow-level prevalence was 1.2%. These figures are greatly lower than the reported DD prevalence in the present study. Pasture-based dairy herds have been frequently reported to be at lower risk of developing DD [38] which could explain the variation in the prevalence estimates. Studies performed on free-stall dairy herds reported much higher cow-level (20.5–66.4%), herd-level (96.1–97%) and within-herd (0–74.3%) prevalence of DD [39–41] than the present study. It is of note, therefore, that our reported within-herd prevalence of DD was somewhere in between the previously reported estimates in pasture-based and free-stall housed dairy cattle. The variation in prevalence estimates could be due to differing management practices and/or environmental factors such as THI.

Lameness, injuries to the hocks and body hygiene have been frequently used as indicators of dairy cattle welfare [42, 43]. The cow-level prevalence of hock lesions (12.9%) was much lower than previously reported (39–68%) in studies that surveyed free-stall and tie-stall housed dairy herds [44, 45]. It is frequently reported that cattle housed on free-stall barns are at significantly higher risk for developing hock injuries [46, 47] and this could explain the reason why the single herd in our study population that had a closed free-stall barn experienced hock lesions in 50% of the examined cows.

In the present study, most cows had a poor hygiene score. The fact that cows in the study population were housed in loose housing barns with sand bedding that is changed once or twice a year depending on the amount of precipitation may have resulted in a lower prevalence of hock lesions and higher prevalence of poor cow hygiene. Studies on cleanliness in different housing systems have shown that cows housed on straw-bedded packs are dirtier than those in cubicle housing but had fewer skin lesions [48, 49] which is consistent with the finding reported here.

Although visual mobility scoring is commonly used to quantify the level of lameness in dairy herds [13–15], it has inherent shortcomings as it is sensitive to intra- and inter-rater variability [50]. In addition, it is labour-intensive and time consuming to perform, especially with increasing sizes of dairy herds. Several studies, however, have reported moderate to good inter-and intra-observer agreement for visual locomotion scoring [50–53]. Automated lameness detection such as the use of accelerometery, force pressure platforms and vision-based methods including video analysis and image processing have been evaluated [54–56]. The overall aim of automated lameness detection methods is to promptly identify and treat lame cows which have been reported to be associated with reduced duration and prevalence of lameness and improved production and welfare outcomes [10, 11]. These technologies have been dependent on reliable visual mobility scoring for initial validation and some studies reported that visual locomotion scoring conducted by trained veterinarians might outperform automated locomotion scoring [57]. In the present study, visual locomotion scoring was performed by the same investigator throughout the study to ensure consistency.

In this study, cows’ hind feet were examined in the milking parlour to diagnose and score DD lesions following washing with water from a hose and using a flashlight. Although examination of the cows in the trimming chute is the gold standard method of identifying and scoring DD lesions [40], the method is costly, labour and time intensive and impractical for regular monitoring of the herd prevalence [58]. The importance of prompt diagnosis and treatment of DD lesions to improve outcomes and to control DD has led to several studies evaluating the agreement between the examination of cows in the trimming chute and in the milking parlour. Solano et al. [40] compared the examination of cows’ hind feet in the milking parlour following washing with water with the use of a mirror and a headlight to examination in the trimming chute. They reported similar overall DD prevalence between the two methods but noted that 51% of active lesions were misclassified as inactive lesions when examined in the milking parlour. Another study that examined the sensitivity of using a mirror without washing and a flashlight to identify DD lesions in the milking parlour reported 90% and 82% sensitivity and specificity to detect DD lesions respectively. However, the sensitivity was reduced to 55% when scoring M2 lesions [59]. A third study that investigated the utility of using a commercial borescope for the diagnosis of DD lesions in the milking parlour without washing compared with direct observation in a trimming chute reported comparable sensitivity and specificity to identify DD-positive cows. However, when DD lesions were dichotomized to active (M1, M2, M4.1) and inactive lesions (M3, M4), the sensitivity of the borescope greatly reduced [60]. Studies have also reported that the examination of cows’ hind feet to identify DD lesions without washing was significantly less sensitive to detect lesions than examination after washing [61, 62]. Taken together, examination of DD lesions in the milking parlour, although not the ideal method for diagnosis of DD lesions, offers the advantages of prompt diagnosis and treatment of the condition and has good sensitivity to differentiate between DD-positive and DD-negative cows [58]. Furthermore, use of this method routinely on the farm could increase the overall sensitivity [60]. In addition, washing of the hind feet should always be performed before any attempt to identify DD lesions. Although, two investigators (the second and the last authors) evaluated cattle for the presence of DD M-scores in the present study, inconsistency between these two observers is unlikely, as many studies reported excellent interobserver agreement for DD M-scores [40, 63, 64].

Another limitation of the present study is that this was a cross-sectional study where the reported prevalence estimates could either reflect high disease incidence with rapid resolution or low incidence with prolonged recovery. A longitudinal study could provide a better picture of the dynamics of lameness and DD in the study population. Furthermore, the selection of dairy farms was not random. We initially aimed to recruit a random sample of dairy farms but there was lack of willingness of many farm owners/managers to participate in the study. This may have resulted in a lack of generalisability of the results of the present study to other dairy herds in Egypt. However, we believe that this would have minimal impact on the study results as our sample was nearly exhaustive.

## Conclusion

This is the first nation-wide study in Egypt to investigate the prevalence of lameness, DD, hock lesions and cow hygiene in dairy cattle herds. The study reported greater lameness prevalence and highlighted the need for implementing measures to reduce the impact of lameness on the dairy industry in Egypt. A moderate prevalence of DD is reported, which corroborates with the management practices of dairy cattle populations in Egypt. The high prevalence of poor cow hygiene throughout the visited farms highlights the need for implementing measures to improve cow cleanliness which is important for milk hygiene and udder health.

## Methods

This was a cross-sectional study that was designed to provide estimates of the prevalence of lameness and DD in dairy cattle herds in Egypt. Information about management and biosecurity practices and potential risk factors for lameness and DD were collected but will be reported separately. The study protocol was reviewed and approved by Zagazig University Veterinary and Agricultural Research Ethics Committee (ZU-IACUC/3/F/147/2021) and informed verbal consent was obtained from all participating dairy farms in the study. Results of the visit were discussed with the farm veterinarian/manager and recommendations about lameness and DD prevention was given.

### Study population and sample size calculation

The target population of the study was the dairy cattle population in Egypt, while our source population was all dairy cattle operations in Egypt milking at least 50 cows at the time of the visit. Sample size calculations were performed using the following assumptions: an expected prevalence of cows diagnosed with a lameness mobility score ≥ 2 of 30%, a precision level around the prevalence estimate of 5% and 95% CI. The following equation was used to estimate the required sample size: $n = {Z^2}\left[ {p\left( {1 - p} \right)} \right] \div {L^2}$; where n is the sample size, *Z* is the *Z*-value reflecting the desired level of confidence (equals 1.96 at 95% confidence level), *L* is the desired precision and *p* is the expected proportion of lame cows. This resulted in a sample size of n = 323 cows. Given the clustering (farms and not individual cows were used as a sampling frame) of our sample, the calculated sample size was adjusted for clustering using the following equation: $N=n (1+\rho (m-1)$; where N is the new/total sample size, n is the original sample size estimate, *ρ* (Rho) is the intra-cluster correlation coefficient and m is the number of cows sampled per herd [65]. Using a *ρ* value of 0.2 [66] and an average number of milking cows per herd (*m*) of 250 cows, the total sample size *N* was estimated to be 16,408. We aimed to recruit 65 dairy farms to achieve the required sample size (i.e., 16,408/250).

### Recruitment

Due to a lack of dairy establishment registration in Egypt, we used several resources to obtain the contact details of eligible dairy farms. These included animal health pharmaceutical companies, dairy technical support departments at milk processing companies and dairy herd consultants. Communication with these parties advised that there are about 300 dairy cattle herds in Egypt. For example, the website of the Juhayna Food Industries company (one of the largest milk processing companies in Egypt) stated that they have been working with 110 dairy farms [67]. We managed to establish a list of contact details for 165 eligible dairy farms. We planned to randomly select 65 dairy farms to achieve the required sample size, but because of a lack of compliance of most of the dairy farms and the need for several contact attempts to convince the farm manager/owner to participate in the study, a more convenient sample of farms was recruited into the study. The farm managers/owners who consented to participate in the study were asked to provide verbal consent.

### Lameness assessment

All lactating cows on each farm underwent mobility scoring on their exit from the milking parlour using a 4-point mobility scoring system [68]. Cows scored ≥ 2 were considered clinically lame and used to calculate the within-herd lameness prevalence. Table 1. provides a description of the lameness scoring system used in the study. Mobility scoring on all farms was performed by the same investigator, to ensure consistency of the results.

**Table 1:** Mobility scoring system described by the Agriculture and Horticulture Development Board [68]

Score	Description
0	“Walks with even weight bearing and rhythm on all four feet, with a flat back. Long, fluid strides possible”
1	“Steps uneven (rhythm or weight bearing) or strides shortened; affected limb or limbs not immediately identifiable”
2	“Uneven weight bearing on a limb that is immediately identifiable and/or obviously shortened strides (usually with an arch of the centre of the back)”
3	“Unable to walk as fast as a brisk human pace (cannot keep up with the healthy herd). Lame leg easy to identify–limping; may barely stand on lame leg/s; back arched when standing and walking”

### Digital dermatitis assessment

Clinical evaluation of the hind feet of all lactating cows on each farm was performed in the milking parlour. The cow hind feet were washed either before or after the milking equipment had been attached to the udder, depending on the milking practice within the parlour. For example, on farms that practice teat washing before the attachment of milking equipment, the parlour workers were asked to wash the cows’ hind feet simultaneously. A flashlight was used to better identify the DD lesions [41]. DD lesions were classified using M-score (M1–M4.1) [69, 70] (Table 2).

A cow was considered DD-positive if it was identified with a DD lesion (M1–M4.1) in at least one of its hind feet and DD-negative if it had normal skin of the hind feet (M0). Cows identified with superficial dermatitis lesions (mild dermatitis around the claws without typical DD lesions) were also considered DD-negative. Only the hind feet had been examined, as previous studies reported that greater than 90% of DD lesions were identified in cows’ hind feet [17, 71, 72].

**Table 2:** Digital dermatitis M-score and descriptors [69, 70]

M-stage	Descriptor
M0	“No sign of pre-existing lesion. Normal skin”
M1	“Small (< 2 cm across) focal active state. Circumscribed lesion”
M2	“Larger (> 2 cm across) ulcerative active stage. Can be painful on manipulation”
M3	“Healing stage. The ulcerative surface is transformed to a dry brown, firm rubbery scab. No pain on manipulation”
M4	“Chronic stage. Surface is raised by tan, brown, black, rubbery, irregular, proliferative hyperkeratotic growths that vary from papilliform to mass-like projections”
M4.1	“Chronic stage with small active M1 focus”

### Hock lesion scoring

The hocks of all milking cows were scored in the milking parlour using the Hock Assessment Chart for Cattle developed by the Cornell Cooperative Extension (Cornell University, Ithaca, NY). A pictorial description of the hock score is available online [73]. Cows identified with normal skin and absence of missing hair had a score of 1, cows showing bald areas on the hock had a score of 2, and cows with evidence of swelling and/or a lesion through the skin had a score of 3. If multiple lesions were recorded on a cow, only the worst hock lesion was considered [74].

### Cow hygiene scoring

Cow hygiene was evaluated using a 4-point score developed by the University of Wisconsin-Madison (Wisconsin, USA). A pictorial description of the score is available online [75]. Each milking cow is given an overall score based on the cleanliness of lower leg, udder, and upper leg to the flank. The proportion of cows assigned to each of the four scores was calculated. In addition, the within-herd prevalence of cows with hygiene score of 3 or 4 was calculated.

### Statistical methods

The data collected included approximate farm locations (latitude and longitude), number of milking cows examined on each farm, and number of cows assigned to each category of the assessment scores (lameness, DD, hock, and hygiene scores). A map of the approximate locations of visited farms was created using QGIS version 3.24 [76]. The prevalence of moderate (lameness score = 2), severe (lameness score = 3) and overall lameness (lameness score ≥ 2) were calculated. Prevalence estimates of DD M-scores and overall prevalence of DD positive cows were calculated. The prevalence of hock lesions was calculated as the proportion of cows identified with a hock score > 1. The prevalence of severe hock injuries was also calculated as the proportion of cows identified with a hock score of 3. Similarly, the proportion of cows assigned to each of the cow hygiene scores was calculated. For all calculations, cow-level, within-herd, and between-herd prevalence and associated 95% CIs were calculated. The prevalence::propCI function in R [77] was used to calculate 95% CIs for prevalence, proportions within individual herds, and the cow- and herd-level prevalence estimates. The Wald method was chosen to calculate these 95% CIs. The average within-herd prevalence estimates and associated 95% CIs were adjusted for clustering within herds using the bootstrap method [78]. All analyses were performed in R software version (4.0.2) [79].

## Data Availability

The datasets generated and/or analysed during the current study are available in the figshare repository https://figshare.com/s/0f74b106271c7e643aa8.
